# Supplementary material for: Detection of Interlayered Illite/Smectite Clay Minerals with XRD, SEM Analyses and Reflectance Spectroscopy
Source: Sensors (Basel). 2022 May 9;22(9):3602. doi: 10.3390/s22093602 (PMC9101579; doi:10.3390/s22093602)
Supplement: Supplementary file 1 [file sensors-22-03602-s001.zip › sensors-1668225-supplementary.pdf]

Table S1. Atom % EDS measurements Scanning Electron Microscope.

	O	*Na*	*Mg*	*Al*	*Si*	*K*	*Fe*	*Cu*
04MRE123-02_pt1	61.11	0.80	0.82	9.38	20.51	6.28	1.11	-
04MRE123-02_pt2	62.62	1.80	1.19	10.10	22.04	2.08	0.18	-
04MRE123-02_pt3	62.97	1.19	1.22	10.36	22.19	1.89	0.20	-
